# Medical Fortnightly

**Published:** 1892-02

**Authors:** 


WE HAVE just received a copy of the Medical Fortnightly, the new St. Louis journal, published by Dr. Bransford Lewis. It is a model of neatness, and presents a corps of collaborators that is sure to win for it general favor.

